# Role of serum TGF-β1 level in atrial fibrosis and outcome after catheter ablation for paroxysmal atrial fibrillation

**DOI:** 10.1097/MD.0000000000009210

**Published:** 2017-12-22

**Authors:** Ye Tian, Yubin Wang, Weijie Chen, Yuehui Yin, Mu Qin

**Affiliations:** a Department of Cardiology, the Second Affiliated Hospital of Chongqing Medical University, the Chongqing Cardiac Arrhythmia Service Center, Chongqing; b Department of Cardiology, Shanghai Chest Hospital, Shanghai Jiaotong University, Shanghai, China.

**Keywords:** atrial fibrillation, atrial fibrosis, recurrence, transforming growth factor-β1

## Abstract

This study aimed to evaluate the relationship between serum transforming growth factor-β1 (TGF-β1) concentration and atrial fibrosis and to determine whether plasma TGF-β1 concentration is an independent predictor of atrial fibrillation (AF) recurrence after catheter ablation.

We included 98 consecutive patients who underwent catheter ablation, including 38 with paroxysmal AF (AF group) and 60 with paroxysmal supraventricular tachycardia (control group). We compared their preablation serum concentration of biomarkers and clinical and echocardiographic findings.

Serum TGF-β1 concentrations, type-III procollagen N-terminal peptides (PIIINP), type-IV procollagen (IV-C), and laminin (LN) were significantly higher in the AF group than in the control group; however, there was no correlation between their concentrations and left atrial diameter (LAD). The area of the low-voltage zone positively correlated with TGF-β1 and PIIINP concentrations, but not with LAD. Atrial tachyarrhythmia (AF and AFL/AT) recurrence was observed in 15 patients (39.4%) at mean 241.4 ± 68.5 days of follow-up 12 months after ablation. Regression analysis revealed that TGF-β1 was a major risk factor for AF recurrence (odds ratio, 1.14; 95% confidence interval, 1.11–1.17; *P* = .02).

Serum TGF-β1 concentration is an independent predictor of AF recurrence in patients with paroxysmal AF and may help identify patients likely to have better outcomes after catheter ablation.

## 1 Introduction

Atrial fibrosis is associated with the electrophysiologic and structural remodeling of atrial tissue; moreover, it is involved in atrial fibrillation (AF) development.^[1]^ Although electrical remodeling can be fully restored several weeks after AF recovery to sinus rhythm, structural remodeling rarely returns to normal and can easily lead to recurrence of AF.^[1–3]^ These findings suggest that compared with electrical remodeling, atrial structural remodeling may play a more critical role in AF recurrence and persistence.

Transforming growth factor-β1 (TGF-β1) is a key cytokine that initiates and promotes the synthesis and metabolism of atrial interstitial collagen. During fibrosis, TGF-β1 can induce over expression of extracellular matrix proteins, resulting in a series of changes in cardiac structure and tissues,^[4–5]^ which consecutively affect electrical conduction, making TGF-β1 essential in regulating atrial interstitial fibrosis. This study investigated the relationship between serum TGF-β1 concentration and atrial structural remodeling. Furthermore, we assessed the efficiency of radiofrequency (RF) ablation therapy for patients with AF by evaluating the relationship between the efficiency of RF ablation therapy and serum TGF-β1 concentration in patients with AF. The study results provide new theoretical and experimental basis for improving the maintenance of long-term sinus rhythm after AF ablation.

## 2 Methods

### 2.1 Participants

This prospective study involved 98 consecutive patients referred for catheter ablation, including 38 patients with paroxysmal AF (AF group) and 60 patients with paroxysmal supraventricular tachycardia (PSVT; control group). Patients with hematologic disease (bone marrow fibrosis, aplastic anemia, and so on), hepatitis (alcoholic hepatitis, viral hepatitis, cirrhosis, and so on), nephritis (acute or chronic glomerular nephritis, nephrotic syndrome, diabetic nephropathy, and so on), neoplastic disorders, recent (<3 months) myocardial infarction or stroke, acute AF precipitated by thyrotoxicosis, or any acute infection were excluded. The study protocol was approved by the Ethics Committee of the second affiliated hospital of Chongqing Medical University, and all subjects provided written informed consent.

### 2.2 Blood sampling and echocardiography

Before catheter ablation, all patients underwent comprehensive echocardiographic examination, and fresh peripheral blood samples were collected. The left atrial diameter (LAD) was derived from the parasternal short-axis view of the transthoracic echocardiogram, and left ventricular ejection fraction (LVEF) were measured using the biplane Simpson method. Serum concentrations of TGF-β_1_, type-III procollagen N-terminal peptides (PIIINP), type-IV procollagen (IV-C), and laminin (LN) were measured by enzyme-linked immunosorbent assays (R&D Systems, Minneapolis, MN).

### 2.3 Catheter ablation for AF

All study patients underwent pulmonary vein isolation (PVI) alone. Details of the ablation procedure have been described.^[6–7]^ Briefly, catheter ablation was performed with the guidance of an electroanatomical mapping system (CARTO, Biosense Webster, CA). The radiofrequency power output was 40 W, the temperature was 43°C, the duration of ablation of each lesion was 20 to 30 seconds, and the saline infusion rate was 20 to 25 mL/minute. Based on the 2006 American College of Cardiology/American Heart Association (ACC/AHA) guidelines, recurrent AF was defined as paroxysmal if the arrhythmia terminated spontaneously. AF was defined as persistent when sustained for longer than 7 days.

### 2.4 Follow-up of AF recurrence

After discharge, patients were prescribed antiarrhythmic drugs for 8 weeks to prevent any early recurrence of AF. All patients were initially followed up 2 weeks after catheter ablation, and then every 1 to 3 months thereafter, either at our cardiology clinic or by referring physicians. Follow-up included 24 hour Holter monitoring or a full week of cardiac event recording. AF recurrence was defined as an episode lasting >1 minute and was confirmed by ECG 3 months after the ablation (blanking period). The end-point for follow-up was the clinically documented recurrence of atrial arrhythmias or repeat ablation procedures.

### 2.5 Statistical analysis

Continuous variables were expressed as mean ± standard deviation and compared using independent samples *t* tests or nonparametric test, whereas discrete variables were expressed as percentages and compared using *χ*^*2*^ tests. One-way ANOVA was used for multiple comparisons of normally distributed data. All tests of significance were two-sided, with a probability value *P* < .05 considered significant. All statistical analyses were performed using SPSS version 18.0 and Graphpad software.

## 3 Results

### 3.1 Patient characteristics

The patient characteristics are shown in Table 1. Of the 98 subjects, 38 (38.8%) with a mean age of 56.8 ± 15.6 years had paroxysmal AF and 60 (61.2%) with a mean age of 54.3 ± 15.8 years had PSVT. Follow-up results 12 months after RF ablation showed that in 23 (60.5%) of the 38 patients with paroxysmal AF, sinus rhythm was restored. The remaining 14 (36.8%) patients experienced AF recurrence, and 1 (2.6%) patient had atrial tachycardia. There were no significant differences in age, LAD, and LVEF in the AF and control groups. However, TGF-β1, PIIINP, IV-C, and laminin serum concentrations were significantly higher in the AF group than in the control group (Table 1).

**Table 1:**
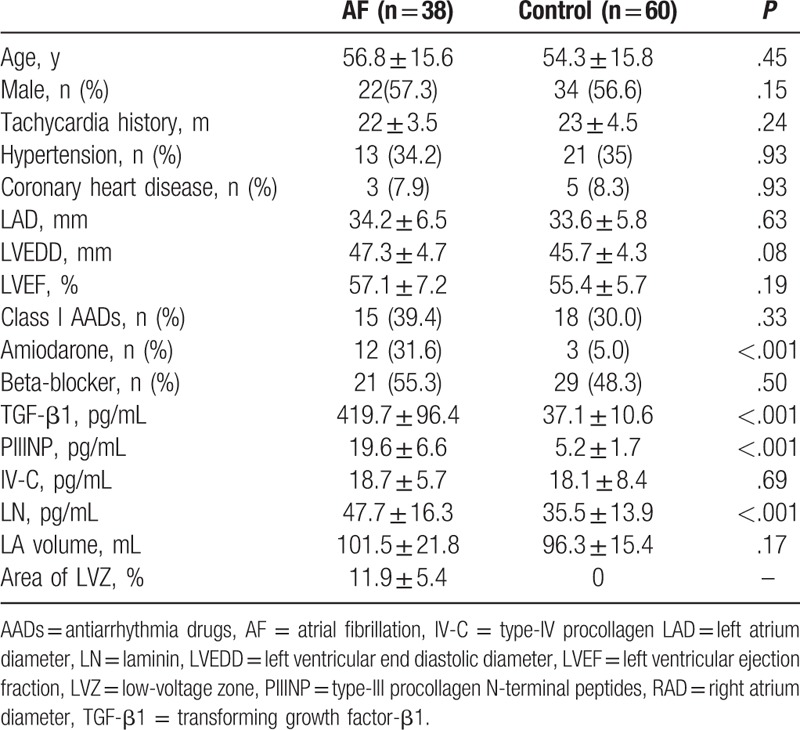
Baseline characteristics.

### 3.2 Serum biomarkers and atrial fibrosis in AF patients

**Figure 1:**
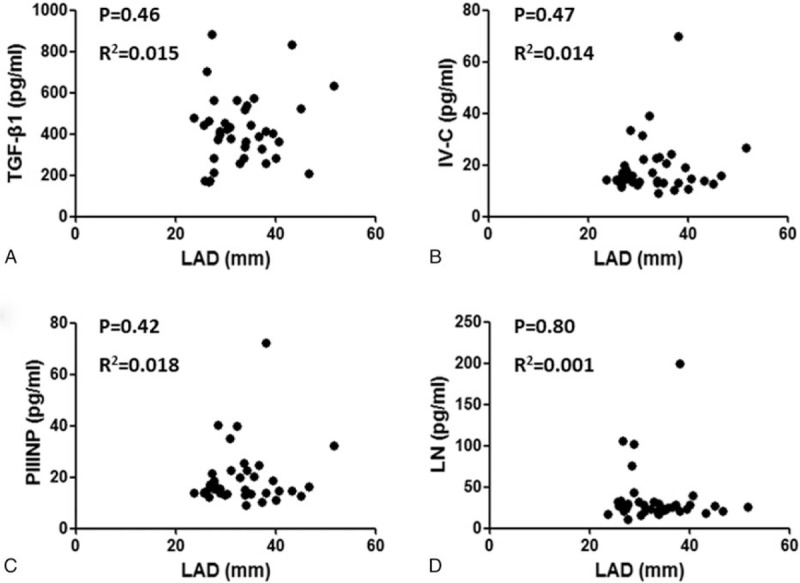
The correlation analysis between left atrial diameter and serum parameters including TGF-β1, PIIINP, IV-C, and laminin.

**Figure 2:**
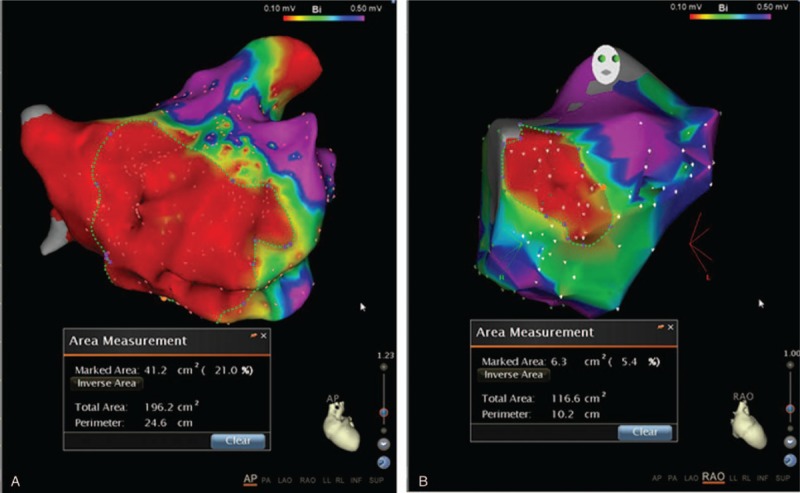
The atrial low-voltage zone was determined by high-density mapping in left atria during procedure. The maximal and minimal low-voltage zones were 21% (A) and 5.4% (B), respectively, in patients with recurrent atrial fibrillation.

**Figure 3:**
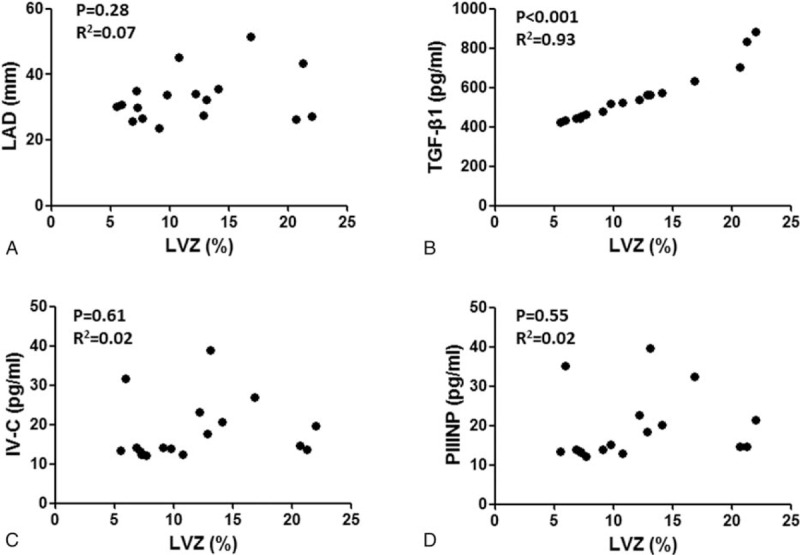
The correlation analysis between atrial low-voltage zone and left atrial diameter (A), TGF-β1 (B), IV-C (C) and PIIINP (D).

Correlation analysis showed that serum TGF-β1 (R2 = 0.015, *P* = .46), PIIINP (R2 = 0.018, *P* = .42), IV-C (R2 = 0.014, *P* = .47), and laminin (R2 = 0.001, *P* = .80) concentrations did not correlate with LAD (Fig. 1). In the 38 patients with AF, high-density mapping of the low-voltage zone (LVZ) of the left atrium during ablation procedures revealed that 17 (47.2%) had LVZs, mainly on the anterior wall and roof of the left atrium. The area of the LVZ ranged from 4% to 21% (Fig. 2). Correlation analysis showed that the area of the LVZ positively correlated with TGF-β1 (R2 = 0.93, *P* < .001) concentrations, but not with LAD (R2 = 0.07, *P* = .28) (Fig. 3).

### 3.3 Serum biomarkers and AF recurrence

**Figure 4:**
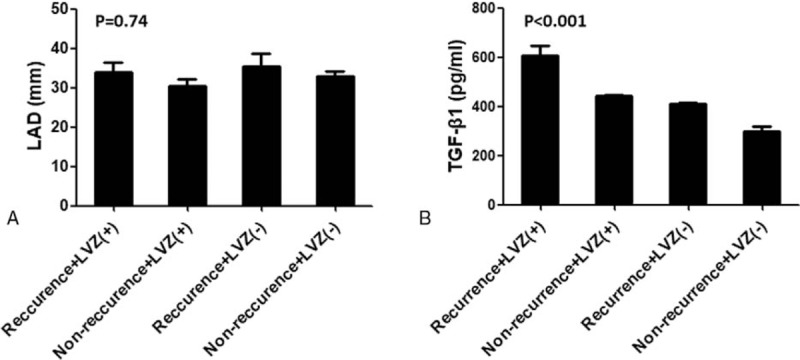
left atrial diameter (A) and serum TGF-β1 concentration (B) according to the low-voltage zone (LVZ) and arrhythmia recurrence. ANOVA analysis shows significant differences in serum TGF-β1 concentration among 4 groups. LVZ (+), patient with atrial LVZ; LVZ(–),patient without atrial LVZ.

Atrial tachyarrhythmia (AF and AFL/AT) recurrence was observed in 15 patients (39.4%) at mean 241.4 ± 68.5 days of follow-up 12 months after ablation. Serum TGF-β1 and PIIINP concentrations were significantly higher in patients with AF recurrence than in those without AF recurrence. By contrast, serum Type-IV collagen and laminin concentrations did not significantly differ in these 2 subgroups. Combining LVZ levels and recurrence, LAD was not significantly different by ANOVA analysis among the 4 groups (*P* > .05) (Fig. 4A). However, serum TGF-β1 concentrations showed significant difference (*P* < .001). In patients with concomitant recurrence and LVZ (+), serum TGF-β1 concentration was significantly higher than that in other groups by Tukey's multiple comparison test (*P* < .05) (Fig. 4B). Potential risk factors that may affect recurrence, including age, LAD, LVEF, LVZ, and serum TGF-β1, PIIINP, Type-IV collagen, and laminin concentrations, were entered into multivariate logistic regression analysis, and it revealed that only serum TGF-β1 concentration was the independent risk factor for recurrence (odds ratio, 1.14; 95% confidence interval, 1.11–1.17; *P* = .02) (Table 2).

**Table 2:**
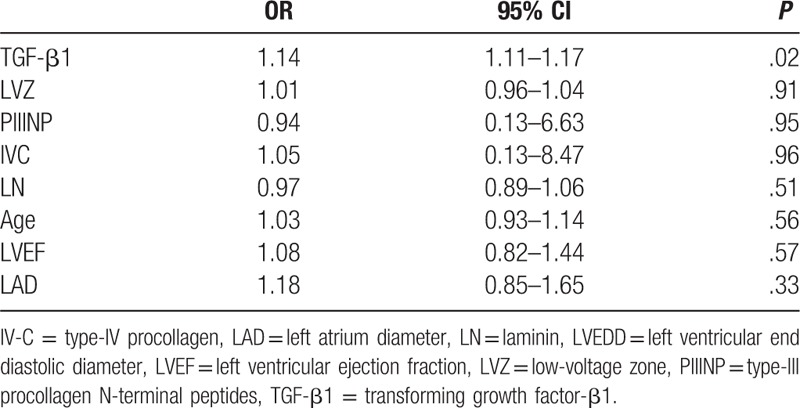
Multivariate analysis.

## 4 Discussion

Several blood markers have been associated with AF in many situations. The serum concentrations of inflammatory biomarkers such as C-reactive protein and IL-6 and red cell distribution width in the preablation phase have been indicated in AF occurrence and recurrence.^[8–9]^ The underlying mechanisms were all related to oxidative stress and chronic inflammation in atrial myocardium, demonstrating that an increase in the concentration of biomarkers may aggravate atrial remodeling.^[9]^ Atrial remodeling is a major characteristic of AF, which provides an inhomogeneous environment for electrical propagation and triggers ectopic or re-entrant activity. TGF-β1 plays essential roles in atrial remodeling process such as tissue repair and fibrosis formation and is considered a key factor causing atrial fibrosis. Examination of the atrial specimens of patients undergoing cardiac surgery showed that TGF-β1 was selectively involved in the formation of atrial fibrosis, through TGF-β1 receptors 1 and 2 and the classical SMAD signaling pathway.^[10]^ Moreover, differences between atrial and ventricular fibrosis reactions may be caused by differences in the ability of TGF-β1 to bind to its receptors or differences in phosphorylation level. Taken together, these findings indicate that atrial fibrosis is an important part of AF occurrence and substrate maintenance. For TGF-β1 to become a potential new therapeutic target, greater understanding of the specific pathways in this process is required.

TGF-β1 may be a cause of AF, inducing and maintaining AF by initiating atrial fibrosis.^[11]^ Collagen, TGF-β1 mRNA, and plasma TGF-β1 concentrations gradually increase in subjects with sinus rhythm, paroxysmal AF, and persistent AF, suggesting that plasma TGF-β1 concentration positively correlates with the degree of atrial fibrosis.^[12]^ Studies in patients with nonvalvular AF have shown that high plasma TGF-β1 concentrations are indicative of low LA endocardial voltage.^[13]^ For example, a study measuring the degree of fibrosis in LA tissue of patients with persistent AF who underwent open heart surgery for valvular heart disease found that plasma TGF-β1 concentrations correlated with the degree of fibrosis.^[14]^ Moreover, preoperative plasma TGF-β1 concentrations were found to predict the persistence of AF 1 year after surgical maze procedures. Taken together, these findings suggest that TGF-β1 is involved in the mechanisms of atrial fibrosis and AF pathogenesis and that plasma TGF-β1 concentration may be a surrogate marker of atrial fibrosis. However, plasma TGF-β1 concentrations are also affected by noncardiogenic factors, such as the kidneys, lungs, and liver. We therefore measured serum TGF-β1 concentration as an indicator of the level of atrial fibrosis in patients with AF. We found that serum TGF-β1 concentration was significantly lower in patients with AF without recurrence than in those with recurrence, suggesting that serum TGF-β1 concentration correlates with AF recurrence. Because this study excluded patients with underlying diseases characterized by severe fibrosis in other organs, the serum TGF-β1 concentrations observed in the study cohort likely reflected the varying degrees of atrial fibrosis in patients with AF.

This study also showed that serum TGF-β1 concentration significantly correlated with the area of LVZ, but not with LA size.^[15–16]^ Previous studies have confirmed the relationship between LA size and AF prognosis. However, the predictive value of LA size has not been consistent across studies,^[17–19]^ which may result from differences in study populations and strategies of catheter ablation. For example, a previous study showed that AF recurrence rates were 85.2% in patients with large LAD and 60% in patients with small LAD and high plasma TGF-β1 concentration, suggesting that LAD alone is not predictive of AF recurrence.^[20]^ The current study, finding that serum TGF-β1 concentration, but not LAD, correlated with left atrial fibrosis, indicates that local atrial fibrosis does not result in LA enlargement. Therefore, serum TGF-β1 concentration and area of LVZ are better predictors of AF recurrence than LAD.

## 5 Limitation

First, nonparoxysmal AF was not mentioned in current study; it is usually associated with structural heart diseases. The underlying process, such as heart failure, may lead to both atrial fibrosis and increased systemic TGF-β1 concentrations. Therefore, TGF-β1 may not be the cause of recurrence but may only serve as a marker that reflects either AF recurrence or its underlying process. Second, serum TGF-β1 concentrations during follow-up were not examined; therefore, further studies are needed to demonstrate the cause-and-effect relationship between TGF-β1 and AF recurrence. Third, although the atrial voltage mapping was performed in current study, there was lack of data on cardiac magnetic resonance to requantify atrial fibrosis. Fourth, although the current study used LAD as a parameter of LA size, the left atrial volume index has been proposed as a potentially better index of LA enlargement as LA remodeling is associated with a reduced sphericity. It may represent an integrated assessment of left atrial chamber, obviating the need to rely on point Doppler estimates that are subject to loading variations.^[21]^

## 6 Conclusion

This study found that TGF-β1 was an independent predictor of AF recurrence and had incremental predictive value over LAD alone. A combination of preablation plasma TGF-β1 concentrations and LAD in patients with paroxysmal AF may help identify patients likely to better respond to catheter ablation.
